# Comparison of the Analgesic Effect of Subcutaneous Bupivacaine Infiltration and Intravenous Diclofenac vs. Intravenous Diclofenac Monotherapy After Inguinal Hernioplasty: A Retrospective Study

**DOI:** 10.7759/cureus.28312

**Published:** 2022-08-23

**Authors:** Said S Alijla, Fitreena A Binti Amran

**Affiliations:** 1 Department of Anaesthesiology, Algerian Hospital, Gaza, PSE; 2 Advanced Medical and Dental Institute, Universiti Sains Malaysia, Pulau Pinang, MYS; 3 Department of General Surgery, Universiti Sains Malaysia, Pulau Pinang, MYS

**Keywords:** mesh repair, postoperative pain relief, local anesthesia, anesthetics, analgesics, herniorrhaphy, diclofenac, bupivacaine

## Abstract

Background

Postoperative pain is a significant problem encountered by patients after a surgical intervention, and there is a crucial need for effective postoperative pain control. The studies have shown that multimodal analgesia and wound infiltration are effective ways to reduce this pain and have a substantial role in the reduction of postoperative medications requirement. This study aimed to evaluate the effect of subcutaneous infiltration of bupivacaine hydrochloride and intravenous (IV) diclofenac as postoperative pain relief in adults undergoing inguinal hernia repair.

Methods

A single-center retrospective study included 104 patients aged 18-65 undergoing unilateral inguinal herniorrhaphy at the selected hospital. The patients were in two groups of 52 each. Group A received a 75 mg dose of IV diclofenac plus a subcutaneous injection of 10 mL of bupivacaine hydrochloride (HCl) 0.5% while Group B only received the IV diclofenac without the bupivacaine injection. The postoperative pain was assessed at one, two, three, six, and 12 hrs after the operation using the visual analog scale (VAS), which exhibited a range of pain from zero (no pain) to 10 (extreme pain).

Results

Of a total of 104 patients, 92% of patients were male. The patients' mean age was 36 ± 11 years, and the mean body mass index (BMI) was 22 ±3 kg/m^2^. American Society of Anesthesiologists physical class I was similar in both groups (90.4% versus 84.6%). Patients in Group A had significantly lower VAS pain scores at one, two, three, six, and 12 hrs after the operation and a longer emergence time than Group B (all *p-*values were < 0.001). Subcutaneous bupivacaine infiltration and IV diclofenac were also found to be an effective analgesic technique in open hernia repair with mesh (*p*-value < 0.001 for all).

Conclusion

Subcutaneous injection of bupivacaine combined with IV diclofenac provides superior analgesia to monotherapy intravenous diclofenac after inguinal hernia repair.

## Introduction

Postoperative pain is the most common adverse event encountered by patients after surgery. Almost 80% of patients experience postoperative pain after surgical intervention, with 75% complaining of pain after receiving a single dose of analgesic [1]. The pain ranges from moderate to severe and is associated with several consequences that affect the patient's physiological and psychological status. Acute pain often induces fear and anxiety, resulting in behavioral, autonomic, and neuroendocrine changes [2].

Inguinal hernia repair is a common surgery in all ages worldwide, with thousands of people seeking treatment for surgical hernia repair to relieve abdominal pain and limitations in physical activity. However, due to postoperative pain, symptoms frequently persist after surgery [3]. Moreover, postoperative pain may delay hospital discharge, loss of appetite, depression, aggression, tissue catabolism, immunosuppression, poor health, and hyperalgesia [4]. Therefore, effective postoperative pain management is imperative, and understanding postoperative pain from the patient's perspective is essential for healthcare professionals to identify ways to improve care. This includes identifying the best analgesic mode and an efficient postoperative pain management approach that may prevent this pain phenomenon and its sequels.

With the advances in perioperative pain management, multimodal analgesia has become essential for pain control [5]. Multimodal analgesia is a combination of two or more analgesics from different classes. Recent theories suggest the addition of local wound infiltration to the analgesic procedure to reduce postoperative pain and the incidence of postoperative chronic pain resulting from the surgical incision. Studies have shown that surgical wound infiltration is effective in reducing postoperative pain in abdominal surgeries, breast surgeries, gynecological surgeries, radical prostatectomy, laminectomy, laparoscopic cholecystectomy, hallux valgus surgery, and herniorrhaphy [6]. Surgical site infiltration with local anesthetic shortens the duration of hospital stay, delays the morphine rescue, reduces consumption, contributes to early recovery after surgery (ERAS), and increases patient satisfaction, although when used in combination with NSAIDs [7]. The hypothesis of the study is tailored to observe whether subcutaneous infiltration with 10 mL of 0.5% bupivacaine hydrochloride (SBI) in addition to intravenous (IV) diclofenac is more effective than simple administration of IV diclofenac for the management of postoperative pain in inguinal hernia repair.

## Materials and methods

A single-center retrospective study was conducted in the department of General Surgery, Algerian Hospital, Gaza, Palestine. The data of 104 patients undergoing inguinal hernia repair were obtained from the hospital data records through the period from September 2020 to August 2021. The hospital officer-in-charge chose patients in both groups according to the medications they received intraoperatively, one case per one control. Elective patients undergoing only unilateral inguinal hernia and aged between 18 and 65 with American Society of Anesthesiologist (ASA) physical health status classifications I and II were included in this observation. Patients with bilateral inguinal hernia and those who did not receive the previously mentioned drugs intraoperatively were excluded from the study.

Demographic data (patient's age, weight, height, body mass index (BMI), and marital status), in addition to clinicopathological data, i.e., chronic diseases, previous surgery, ASA, type of surgical repair, as well as the time of anesthesia and surgical procedure were collected. Furthermore, because this project was a retrospective medical record review and quality improvement study, patient-informed consent was not required, and ethical approval was granted under the supervision of Universiti Sains Malaysia with the protocol code USM/JEPeM/21110757. The study was conducted with proper permission from the hospital.

All procedures were performed by the same surgical team at the Algerian hospital. All patients received general anesthesia. Fentanyl citrate (1.5-2 mcg/kg, IV), propofol (2 mg/kg, IV), and atracurium besilate (0.2 mg/kg, IV) were used for induction of general anesthesia, and a 50% mixture of oxygen/nitrous oxide and isoflurane 1.15% (1 MAC (minimum alveolar concentration) were used for maintenance. Patients in Group A (n= 52) received a 75 mg dose of intravenous diclofenac, and intraoperatively, a single shot of 10 mL of 0.5% bupivacaine HCl was subcutaneously administered beneath the incision margins after the closure of the skin layer, whereas Group B (n=52) only received 75 mg of intravenous diclofenac. The intravenous diclofenac was given slowly in a solution for up to 20 mins.

At the end of the surgery, patients were transferred to the post-anesthesia care unit (PACU), and the postoperative pain score was assessed using VAS (0-10) at one hr and two hr. Then, patients were transferred to the ward, and the VAS score was assessed at three, four, six, and 12 hrs postoperatively. Patients who submitted a VAS pain score of 3 or above have received painkillers accordingly. Examples of pain control regimens followed in the hospital are intravenous acetaminophen, diclofenac, ketorolac, and meperidine as needed. At discharge, scheduled oral analgesics like acetaminophen 1 gr, ibuprofen 400 mg, or diclofenac 50 mg were prescribed as part of the postoperative pain control regimen.

Outcome measures

The primary purpose of this study was to evaluate the effect of subcutaneous bupivacaine HCl infiltration added to the pain control regimen. The secondary outcome includes the effect of SBI on postoperative pain in the presence of the mesh repair technique. The average pain scores in the PACU and surgical wards were obtained using VAS, which exhibited a range of pain from zero (no pain) to 10 (extreme pain).

Statistical analysis

Statistical analysis was done using SPSS® (Statistical Package for the Social Sciences) version 26.0 (IBM Corp., Armonk, NY). Data were demonstrated as means with standard deviation and counts with percentage as appropriate. Continuous data, such as age, BMI, anesthesia duration, and surgery duration, were analyzed using the dependent t-test. The associations in categorical data, such as gender, ASA physical health status classification, and type of surgical repair, were determined using the chi-square test or Fisher's exact test where applicable. A p-value < 0.05 was considered statistically significant. The postoperative pain score was analyzed using the Mann-Whitney test. All the tests were two-tailed.

Study size calculation

The highest sample size calculation based on general and specific objectives is 104 participants. This is using specific objectives, for example: Identify the postoperative pain of a patient undergoing inguinal herniorrhaphy within six to 12 hours after the operation.

Sample size independent t-test

Based on the paper "Local Wound Infiltration with Ropivacaine for Postoperative Pain Control in Caesarean Section" [8].

## Results

Of a total of 280 patients who had undergone inguinal hernia repair from 2020-2021, only the data of 104 patients were obtained from the hospital records. Ninety-six patients were male (92.3%), and eight were female (7.7%). The patients' mean (SD) age was 36 ± 11, and the mean BMI was 22 ± 3. ASA physical health status class I was similar in both groups (90.4% versus 84.6%), and the differences were statistically insignificant (p = 0.38). The patients were divided into two equal groups of 52 each. Group A received SBI in addition to IV diclofenac. Group B received only IV diclofenac monotherapy. Both groups were well-matched in the most basic characteristics such as gender, age, BMI, duration of surgery, and ASA classification. However, there was a statistically significant difference in the duration of anesthesia and the median anesthesia duration for SBI with IV diclofenac and IV diclofenac monotherapy was 72 ± 13 and 61± 7 mins, respectively. This is due to the need to perform and administer local anesthesia postoperatively. The descriptive characteristics of the study groups are summarized in Table 1.

**Table 1:** Descriptive characteristics of the study groups SD = Standard Deviation, BMI = Body Mass Index, SBI = Subcutaneous Bupivacaine Infiltration, IV = Intravenous, p-value 0.05 = Significant Difference, χ2 = Chi-Square Value * Statistically significant at p-value <0.05. ** Statistically significant at p-value <0.001

Variable	Total n (%) or (Mean ± SD) n=104	SBI plus IV Diclofenac Group n (%) or (Mean ± SD) n=52 (50%)	IV Diclofenac Monotherapy n (%) or (Mean ± SD) n=52(50%)	χ^2^	p-Value
Gender				0.07	0.46
Male	96 (92.3%)	47 (90.4%)	49 (94.2%)		
Female	8 (7.7%)	5 (9.6%)	3 (5.8%)		
ASA class				0.09	0.38
I	91 (87.5%)	47 (90.4%)	44 (84.6%)		
II	13 (12.5%)	5 (9.6%)	8 (18.4%)		
Type of repair				0.21	0.03*
Mesh	81 (77.9%)	36 (44%)	45 (56%)		
Non-mesh	23 (22.1%)	16 (70%)	7 (30%)		
Age (year)	36 ± (11)	33 ± (11)	39 ± (11)		0.01*
BMI (kg/m^2^)	22 ± (3)	22 ± (3)	22.5 ± (3)		0.22
Duration of anesthesia	66 ± (11)	72 ± (13)	61 ± (7)		0.001*<
Duration of surgery	53 ± (10)	54 ± (13)	52 ± (7)		0.19

**Figure 1:**
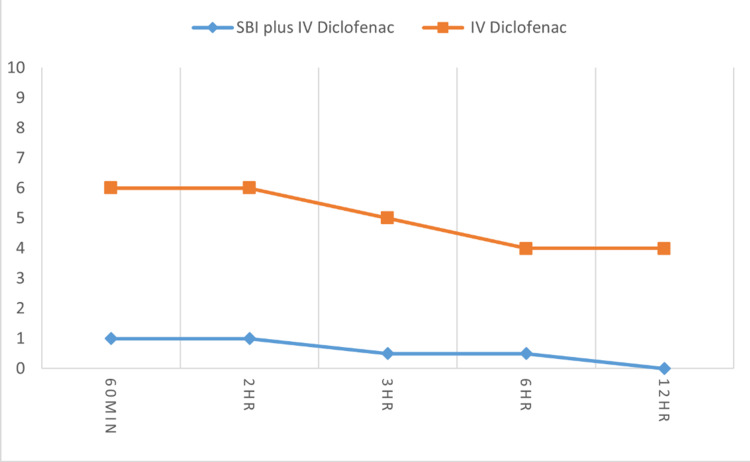
Median postoperative visual analog scale scores

The VAS differences between the groups were statistically significant throughout the five intervals of time (Figure 1). A significant difference in VAS pain scores was detected at one, two, three, six, and 12 hrs after the surgery. The median VAS scores in Group A were significantly lower than those in Group B at the first 12 hours (all p < 0.001). The time to first analgesic demand and postoperative analgesia requirement was significantly different in Group A. The details are shown in Table 2.

**Table 2:** Median pain score among both groups VAS = Visual Analog Scale, SBI = Subcutaneous Bupivacaine Infiltration, Q = Interquartile Range, IV = Intravenous * Statistically significant at p-value <0.001

Variable	Total N = 104 Median (Q1-Q2)	SBI Plus IV Diclofenac N= 52 Median (Q1-Q2)	IV Diclofenac Monotherapy N= 52 Median (Q1-Q2)	P-Value
VAS Score at 1 Hour	2(2-6)	1(0-2)	6(3-8)	<0.001**
VAS Score at 2 Hour	2(1-6)	1(0-2)	6(4-7)	<0.001**
VAS Score at 3 Hour	2(0-5)	<1(0-2)	5(4-6)	<0.001**
VAS Score at 6 Hour	3 (0-4)	<1(0-1)	4(4-6)	<0.001**
VAS Score at12 Hour	2(0-4)	<1(0-1)	4(3-6)	<0.001**
Time to First Analgesic Demand (Hour)	6(1-12)	12(12-12)	1(1-2)	<0.001**
Morphine Equivalent Dose 0-12 hrs (mg)	4(0-4)	<1(0-1)	10(8-10)	<0.001**

The study also noted that out of 104 patients, 81 (77.9%) patients received mesh repair for their inguinal hernia. A comparison of postoperative pain between the two analgesic groups in the presence of a mesh was analyzed, and the analysis was statistically significant. The mean pain score was found to be reduced in Group A compared to Group B. Group A had significantly lower pain scores in the first 12 hrs after the operation with all p-values < 0.001 (Table 3).

**Table 3:** Mean postoperative visual analog scores in the presence of a mesh SD = Standard Deviation, SBI = Subcutaneous Bupivacaine Infiltration, VAS = Visual Analog Scale * Statistically significant at p-value <0.05

Variable	Total n=81 (Mean ± SD)	Mesh repair + SBI and Diclofenac n=36 (Mean ± SD)	Mesh repair + Diclofenac monotherapy n=45 (Mean ± SD)	P-Value
VAS Score at 1 Hour	3± (3)	1± (1)	5 ± (3)	<0.001**
VAS Score at 2 Hour	3± (3)	<1 ± (1)	5 ± (2)	<0.001**
VAS Score at 3 Hour	3± (3)	<1 ± (1)	5 ± (2)	<0.001**
VAS Score at 6 Hour	3± (2)	<1 ± (1)	4 ± (1)	<0.001**
VAS Score at12 Hour	3± (3)	<1 ± (1)	5 ± (2)	<0.001**

## Discussion

Of a total of 104 patients undergoing inguinal hernia repair, 96 (92.3%) were male. KS and Rao found that inguinal hernia is a common surgical procedure that accounts for approximately 75% of all abdominal hernias and the majority of patients were males [9]. Males are 25 times more likely to have inguinal hernia than females; this observation is consistent with our findings. The mean age of patients in our study was 36 ± 11 years, which contradicts the previous studies that showed that the mean patient age was above 49 years [10,11]. To the best of our knowledge, this may allude to the patient's health education level and early engagement with the healthcare system. Moreover, the mean BMI in our study was 22 kg/m^2^, which interprets that patients were at normal body weight. Therefore, our finding is inconsistent with other studies, which indicate that patients with lower BMI are at higher risk of inguinal hernia [12,13].

Inguinal hernia repair is usually performed to avert or reduce the hernia's side effects. However, postoperative pain after inguinal hernia repair remains a significant challenge for healthcare providers, and the need for appropriate postoperative pain control is crucial. Postoperative pain control is the primary clinical and patient-desired outcome issue that needs proper attention and intervention. Efficient postoperative pain management significantly reduces the need for postoperative analgesia and rescue, improves patient satisfaction, allows for early recovery after surgery, improves physical activity, and speeds up hospital discharge [14]. The American Society of Anesthesiologists recommends multimodal analgesia for the management of acute pain because drugs with different mechanisms of action target different pain pathways, which ultimately results in synergism or an additive effect and allows better pain control in addition to a reduction in the drugs' adverse effects [15].

Another study demonstrated that it is essential that surgical site infiltration is combined with other non-opioid analgesics to attain the maximum analgesic effect [16]. Local anesthesia infiltration is a reversible blocking of nerve conduction around their administration site by inhibiting voltage-gated sodium channels at the nerve endings and along the axon, this process results in temporary loss of sensation in the surrounding tissue area, the duration of sensation loss depends on the type of local anesthetic agent used [17]. Bupivacaine HCl (1-butyl-2',6'-pipecoloxylidide hydrochloride) is one of amid group local anesthetics that can be used in the perioperative period to provide an analgesic effect with the longest duration among its staff [18].

In this study, we observed the analgesic effect of SBI and IV diclofenac (A) on postoperative pain relief in patients undergoing inguinal hernia repair compared with a monotherapy diclofenac administration (B). A single shot of subcutaneous bupivacaine infiltration and intravenous diclofenac was given intraoperatively. The results showed statistically significant differences in VAS scores between the two groups. The VAS score in the bupivacaine group was significantly lower compared with the diclofenac group at one, two, three, six, and 12 hrs postoperatively. The lower pain score reflects that the bupivacaine group had a higher satisfaction level and less analgesic consumption. These findings correlate well with the present studies, which demonstrated that bupivacaine infiltration reduces postoperative pain in the first 24 hours of surgery without adverse cardiological or neurological effects [19,20]. The results exhibited that although local infiltration is a useful technique that can be adapted to manage postoperative pain in several surgeries, such as abdominal surgeries (total abdominal hysterectomy, colorectal surgery, cesarean section, and inguinal hernia), laminectomy, radical prostatectomy, laparoscopic cholecystectomy, breast surgeries, and hallux valgus surgery [21,22]. However, a study has shown that wound infiltration with a local anesthetic is ineffective and does not show better efficacy than a placebo in patients undergoing osteosynthesis of extracapsular hip fractures [23].

Surgical wound infiltration is a component of multimodal analgesia that plays a substantial role in the reduction of acute postoperative pain. Andersen et al. [24] observed reduced pain and lower postoperative medication requirement from eight to 98 hours postoperatively, this owes to the intraoperative periarticular injection of local anesthetic followed by intraarticular catheter insertion on Day 1, and a prospective study included 56 patients undergoing open hepatectomy, Sun et al. found reduced pain at the first 12 hrs of surgery and the total sufentanil consumption was significantly lower after ropivacaine infiltration [25].

Additionally, inguinal hernia repair using mesh is the preferred method when the prevention of recurrence is the primary goal. However, mesh implantation is associated with increased complications for patients, particularly postoperative pain, for this reason, some patients request non-mesh hernia repair [26-28]. In our study, we found adding subcutaneous bupivacaine infiltration to the analgesic regimen attenuates the postoperative pain even in the presence of mesh repair. However, this is a retrospective study. Hence, the data collection has limited information, a small sample size, and a short follow-up time. Therefore, a larger prospective study and longer pain follow-up time can be conducted in the future to improve the output of this study.

## Conclusions

The study concludes that subcutaneous infiltration with bupivacaine HCl plus intravenous diclofenac can improve postoperative pain control after inguinal hernia repair and reduce the acute postoperative pain after mesh implantation. SBI and IV diclofenac markedly reduce the total postoperative analgesia consumption. This simple, safe, and effective analgesic technique can be applied in most surgeries to reduce acute postoperative pain.
